# Urban Planning and Health Inequities: Looking in a Small-Scale in a City of Cape Verde

**DOI:** 10.1371/journal.pone.0142955

**Published:** 2015-11-23

**Authors:** Luzia Gonçalves, Zélia Santos, Miguel Amado, Daniela Alves, Rui Simões, António Pedro Delgado, Artur Correia, Jorge Cabral, Luís Velez Lapão, Isabel Craveiro

**Affiliations:** 1 Unidade de Saúde Pública Internacional e Bioestatística, Instituto de Higiene e Medicina Tropical, Universidade Nova de Lisboa, Lisboa, Portugal; 2 Centro de Estatística e Aplicações da Universidade de Lisboa, Lisboa, Portugal; 3 Serviço de Nutrição e Dietética, Centro Hospitalar Lisboa Ocidental EPE – Hospital Egas Moniz, Lisboa, Portugal; 4 GEOTPU - Grupo de Estudos de Ordenamento do Território e Planeamento Urbano, Faculdade de Ciências da Universidade Nova de Lisboa, Costa da Caparica, Portugal; 5 Direcção Nacional da Saúde, Ministério da Saúde, Praia, Cabo Verde; 6 Instituto Nacional de Saúde Pública, Ministério da Saúde, Praia, Cabo Verde; 7 Global Health and Tropical Medicine, IHMT-UNL, Lisboa, Portugal; Örebro University, SWEDEN

## Abstract

**Background:**

The lack of high-quality data to support evidence-based policies continues to be a concern in African cities, which present marked social, economic and cultural disparities that may differently impact the health of the groups living in different urban contexts. This study explores three urban units—formal, transition and informal—of the capital of Cape Verde, in terms of overweight/obesity, cardiometabolic risk, physical activity and other aspects related to the urban environment.

**Methods:**

Quantitative and qualitative research methods were used in this intra-urban study. A proportional stratified random sample (*n* = 1912 adults), based on geographical coordinates of private households, was selected to apply the UPHI-STAT questionnaire. In a second stage (*n* = 599), local nutritionists collected anthropometric measurements (e.g., height, waist circumference) and body composition by bioelectric impedance (e.g., body weight, body fat, muscle mass). In a third stage, pedometers were used to count study participants’ steps on working and non-working days for one week (*n* = 118). After a preliminary statistical analysis, a qualitative study was developed to complement the quantitative approach. Generalized linear models, among others, were used in the multivariate analysis.

**Results:**

Insecurity was the main concern among survey respondents in the three units, notwithstanding with significant differences (*p* < 0.001) among units. About three-quarters (76.6%) of the participants of the informal unit emphasised the need for more security. The formal unit presents an older age structure (61.3% above 40 years old) and the transition unit a younger age structure (only 30.5% above 40 years old). Some health-related variables were analysed in each unit, revealing an excess of chronic conditions reported by inhabitants of informal unit, compared with the formal unit despite the informal unit’s younger age profile. The self-reported hypertension varied significantly among urban units (*p* < 0.001), with 19.3% in the formal unit, 11.4% in the transition unit and 22.5% in the informal unit. Women of the urban units present significant differences (5% level) for body mass index calculated from self-reported measures (*p* < 0.001), fat mass (*p* = 0.005), waist circumference (*p* = 0.046) and waist-to-height ratio (*p* = 0.017). For women, overall physical activity was 67.4% (95%CI [64.8,70.0]), with differences among urban units (*p* = 0.025). For men it was of 85.2% (95%CI [82.3,87.6]), without significant differences among urban units (*p* = 0.266). The percentage of women and men who reported physical activity in leisure time was discrepant, with 95%CI [22.6, 27.4] and [53.2, 60.2], respectively. The results of pedometers also indicated that men walk significantly more than women (*p* < 0.001), with a difference of approximately 2000 steps/day.

**Conclusions:**

The data collection process itself also gave us some clues on the involvement of local communities, exploring the potential of social capital of these settings and the role of the woman in family and society in Cape Verde. The higher participation of women and residents of informal unit (the most disadvantaged groups) suggests these as the priority target groups for health promotion campaigns. The link between health planning, urban planning and security of the city needs to be reinforced to minimize health, social and gender inequalities.

## Introduction

Social and physical environments have been recognized as important determinants of health [1, 2]. In Africa, rapid urbanization has changed disease patterns. Abrahams et al [3], and the references therein, describe three transition processes: the demographic transition (change from a period of high fertility and mortality to a one of low fertility and mortality); the epidemiological transition (shift from a high prevalence of infectious diseases to a one of high prevalence of chronic and degenerative diseases); and the nutritional transition (a change in diet, to energy-dense diets and nutritional imbalance, accompanied by shifts in physical activity patterns). Official statistics show that developing countries undergoing socioeconomic transition face an increase of the chronic diseases and a decline of infectious diseases. Abraharms et al [3] note that a country or a region within a country at any given time may be at different stages of nutritional transition. In particular, the urban characteristics vary widely between cities and within the same city coexist formal (organized, planned, with infrastructures, etc.) and informal settlements (unauthorized, unplanned, lacking infrastructures, etc.), resulting in socio-economic-cultural inequalities that may affect the health of their residents [4]. Understanding the genesis and the shape of the city makes it possible to recognize the diversity of its neighbourhoods and the characteristics of the dwelling and their inhabitants. Expansion of the cities in developing countries, and in particularly Africa, as rural migrants seek access to work and other opportunities has resulted in the growth of informal areas without infrastructures. On the other hand, the process of reconversion of informal areas in order to provide basic services (e.g., water, power and sewage) is expensive and time consuming resulting in the existence of different conditions and opportunities to their population [1]. In the Sub-Saharan African region, it is estimated about 62% of population lives in informal settlements [5].

Worldwide, it is estimated that about one billion people live in informal settlements and slums [1, 6]. Accurate health statistics are almost non-existent in informal settlements and the lack of data has masked health disparities within cities [1, 7, 8]. It is essential that slum dwellers are also captured in health statistics to make it possible to identify and address intra-urban disparities [7]. Because different countries use different specific and dynamic terminology to identify “urban” [4] and “slum” [8], official statistics on urbanization are not sufficient to accurately describe inter-urban and intra-urban variations, and more research studies are needed. Diez-Roux [9] pointed out that there has been an explosion of interest in neighbourhood health effects within public health and epidemiology because it is clearly inadequate to consider only individual-level characteristics; it is necessary also to consider characteristics of the groups, or contexts, where the individuals belong, as well as understand how social inequalities impact health. The role of housing policy and urban planning policy on health, taking into account their impacts on the context in which individuals live and work, has been explored by several authors [6, 9, 10]. At least in developed countries, the role of urban planners and developers in planning of and/or modifying of the built environment to improve health populations and to reduce harmful exposures is well established. Neighbourhood deprivation and inequity in the built environment have been linked to physical inactivity, obesity and cardiovascular diseases, among others, in developed countries [11–13]. In Africa, there is a lack of integration of urban planners to help solve specific health problems despite recent developments that demonstrate the willingness and awareness of planners to deal with health problems in urban settings [14].

According to Ziraba et al [15], overweight/obesity might take epidemic proportions in Africa. Malhotra et al [16] reported that regional and national studies have shown differences in prevalence of overweight and obesity by age and gender, but there are few studies evaluating the association of obesity with socio-demographic factors. Moreover, these studies are based on Demographic and Health Surveys designed to collect nationally data on demographic and health indicators, usually at regular intervals of five years [15]. Abrahams et al [3] described 40 countries in Sub-Saharan Africa and Cape Verde is displayed as having relatively high levels of overweight/obesity, and low levels of underweight in women, as well as high intakes of energy and fat. In South Africa, studies for identifying priority groups for public health obesity control programs have been implemented. In other African countries, these studies are rare.

According to the World Bank, Cape Verde is a lower middle income country. In Cape Verde, to the best of our knowledge, there has been no research on identifying urban health patterns. An epidemiological transition is currently under way in this archipelagic country, where the non-communicable diseases are already the leading causes of death [17]. This work describes a part of a research project, *Urban Planning and Health Inequities—moving from macro to micro statistics* (UPHI-STAT project), which aims to study three urban units/areas—formal, informal and transition—in the city of Praia, using a mixed of quantitative and qualitative research methods to provide critical information for understanding how socio-economic-cultural inequalities and the urban morphology affect the health of their residents. According to its national census [18], in 2010, Cape Verde had 491.875 inhabitants and the municipality of Praia presented a strong growth of its population during the prior two decades: 71.276 in 1990, 106.348 in 2000, and 131.719 in 2010, respectively, with 86.5%, 88.5% and 97.0% living in urban environment. Thus, the city of Praia had around 127.767 inhabitants, being the capital and largest city in Cape Verde. Compared to Nairobi, for example, it is a small African city, where disparities between rich and poor urban areas are not so marked. The informal unit is not a typical slum and the formal unit is not an upscale residential area. Nonetheless, it is important to investigate differences in the health profile of urban units of this small city.

Involvement of residents is pointed out by Unger and Riley [8] as an important step to address social and health disparities (in slums). The concept of social capital is related with norms, trust, and networks that can facilitate collective action for mutual benefit ([19] and the references therein) which can be considered important determinants of health. Tomita and Burns [19] assessed neighbourhood-specific social capital in a South African study, using four variables in the SA-NIDS Household questionnaire: (1) support network and reciprocity, (2) association activity, (3) collective norm and values, and (4) safety. The authors included a question about individual preference to remain in the neighbourhood, which is considered a moderator of the relationship between neighbourhood social capital and health outcomes [19, 20]. With the exception of South Africa and some studies in Kenya (Nairobi) [14, 21], to the best of our knowledge, there are few studies about this topic in African countries, compared with other parts of the world. In particular, the bidirectional relationship between neighbourhood design and social capital was explored in Galway, a small city of Republic of Ireland [22]. The potential of social capital of informal units for action in health is also described by Unger and Lee [8], among other, giving a particular attention to slums of São Salvador da Bahia and Rio de Janeiro in Brazil.

Cape Verde is characterized by a historical grounding of transnational family life contributing to a central role of the women in the family structure (e.g., [23]), frequently labelled ‘matrifocal’ or ‘female-headed’ [24]. The female position in the family was fostered in a context of flexibility of households and instability of conjugal relations, described by several works [23, 24]. In fact, most women in Cape Verde do not live with the father of their children and receive almost no financial or emotional support from them, a reason why most women organize their lives counting on other members of the household and other women living in the neighbourhood. This scenario is the basis for the description of Cape Verde by Drotbohm [24] as a country where female relatives still carry more responsibilities than males. The local notion of family as not necessarily based on biological kinship or bound to a specific locality but having its centre in a certain household (see [24] and references therein) was shaped by the historical development of Cape Verdean culture based on migration [24].

The Commission on Social Determinants of Health [25], and other authors (e.g., [21]), also highlighted the role of the collaboration between civil society and local communities on the one hand, and policy makers and researchers on the other to minimize systematic differences in health of different groups living in urban (and rural) contexts. As pointed out by Ompad et al [26], identifying and addressing disparities in terms of social determinants of health is an important step to achieve the Millennium Development Goals, namely the first three goals related with poverty, education and gender. In urban poor settings, Kjellstrom and Mercado [1] pointed out gender as the major determinant of disadvantage in health.

In the scope of the UPHI-STAT project, the social and physical environments of each unit/area or subarea were described (e.g. basic infrastructure, sanitation, transportation, outdoor areas for exercise and recreation) and their residents were inquired, giving attention to biological and socio-demographic variables, some household possessions and amenities, movements inside and outside areas related with work and access to services and food. The individual data were focused on variables with importance to chronic diseases (e.g. waist circumference, body mass index (BMI), diet, physical activity). The main objectives of the UPHI-STAT project are: (i) to characterize the morphology of the city in terms of social and physical environments (e.g. leisure facility, services, transportation, healthcare units), in the city and in the three urban units; (ii) to characterize each unit in terms of socio-demographic characteristics, physical activity, diet, and biological markers to cardiovascular diseases; (iii) to explore associations and correlations between different types of variables at individual, subarea and area levels; (iv) to identify the health profile of each urban unit; and (v) to provide critical information for understanding how socio-economic-cultural inequalities and the urban morphology affect the health of the individuals of the areas and subareas. The lack of high-quality data to support evidence-based policies continues to be a concern in many African countries, and the UPHI-STAT project can be used as a baseline for future surveillance in Praia. To our knowledge, this intra-urban study is pioneer in Cape Verde. The project was implemented by a multidisciplinary team that combines different areas of expertise, including international public health, nutrition, urban planning, social epidemiology and statistics.

This manuscript will present some findings of the UPHI-STAT project with focus on (i) differences among inhabitants of the three urban units; (ii) how the inhabitants perceive their neighbourhoods; (iii) what were the characteristics of the participants in the different stages of our study; (iv) overweight/obesity and abdominal adiposity indicators with focus on women of the three urban units; (v) physical activity (global, work and leisure) by gender and urban units, using self-reported information and pedometers in working and non-working days; (vi) the relationship between physical activity in leisure time, perception of security and lack of infrastructures. The manuscript also describes the data collection process and explores benefits and drawbacks in terms of methodological issues and the possible involvement of civil society and local communities in future actions. We will give a particular attention to residents of the informal unit and women, because we anticipated they are the most disadvantaged groups, in terms of some health outcomes (e.g., self-reported chronic conditions, obesity, cardiometabolic risk) and some modifiable factors (e.g., leisure time physical activity).

## Materials and Methods

### Studied areas

**Fig 1:**
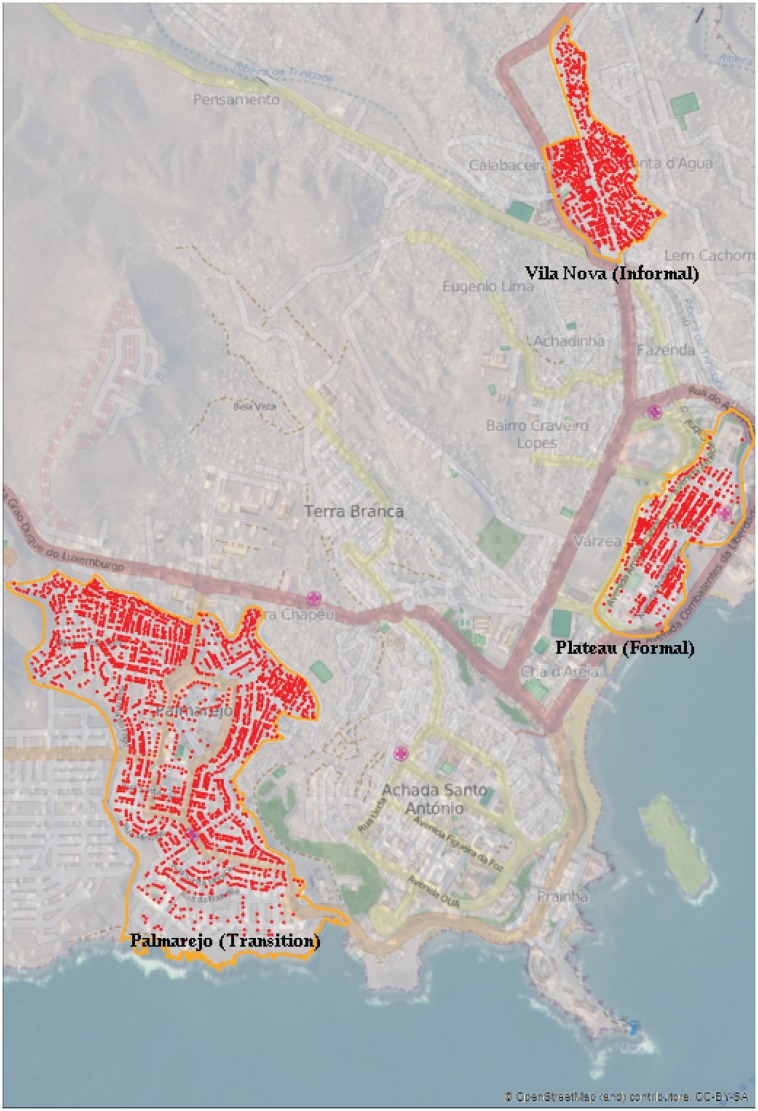
Urban Units with sampling points. Source: http://eol.jsc.nasa.gov/. Figure is similar but not identical to the original image, and is therefore for representative purposes only.

This is an intra-urban study [26] that includes three urban units of the city of Praia, Cape Verde (see Fig 1), corresponding to the following neighbourhoods: Plateau (or Platô)—the formal unit; a part of Palmarejo—the transition unit, and a part of Vila Nova—the informal unit. The three neighbourhood units have different levels of infrastructure and population density. Understanding the urban morphology of the city is based on different analyses: quantitative (density, flow, volumetric coefficients, dimensions, road profiles), organizational and functional (e.g., human activities of living, working, education, trade, leisure) and qualitative (comfort, climate adaptation, accessibility, etc). The formal urban unit reflects a more regulated and consolidated urban form (with water, energy, sewage and waste collection, public key equipment and open spaces). The informal unit is characterized by the absence of basic infrastructures and services with sidewalk and roads in poor conditions, because this area has grown without an effective planning model. This unit has reduced economic activity and the majority of the population has rural origins. The transition unit combines both formal and informal characteristics.

In 2014, Praia’s formal and informal areas comprised about 43% and 21% of the total of the city, respectively [27]. We use “urban unit” or “urban area”, however, this terminology varies across countries. Based on Geographic Information Systems (GIS), the delimitation of each urban unit was performed using the Geographical Urban Units Delimitation (GUUD) model [28] adapted to the context of this city and to the objectives of this research. The GUUD model operates on city macro scale by defining simplest set of urban units, called by “cellular units”, that can be considered homogenous and representative in accordance with the local reality. The model makes it possible to consider a variety of determinants as: urban compactness and morphology, street patterns, zoning, population density, socio-economic characteristics and environmental performance. Statistical subsections joined to the information on geographical data base provided by the INE CV [18] have been adopted as starting point for the urban unit (See Table 1 and Fig 2 in [27]). The statistical subsection has also been adopted as a geo-referenced grid in order to implement this approach to the city of Praia. The use of this geo-referenced grid allows to work with several levels of quantitative information in the research process. From within this geo-referenced grid, the GUUD model is calibrated to collect a selection of data inputs to focus the delimitation criteria: construction timeline; population density; urban morphology and land-use patterns. These frameworks enable one to shift from the macro scale of city to the micro scale of urban unit, filtering the global database with selected parameters. In each urban unit, the geographical coordinates of some infrastructures, services and households, among other, were identified. The geographical coordinates of the private households were explored to provide the sampling frame used in the sampling strategy.

### Sample size, sampling strategies and data collection

Sample size determination, to implement a research project in the field, needs to address statistical requirements and time, cost, human resources, and ethical and technical issues. The sample size required to estimate a binomial proportion (e.g. prevalence of overweight), using a confidence interval with a specified level of confidence and a desired precision, for both an infinite population and for a finite population, is a common problem with several answers, according to desired values, methods and initial estimated proportion [29, 30]. According to WHO [31], in 2008 the estimated prevalence of elevated blood pressure in Cape Verde was 44.1% and the prevalence of overweight was 34.4%. As the first estimate (44.1%) results in a larger sample, in each area, around 592 inhabitants (not corrected by population size—numbers of inhabited households) should be selected to ensure a 95% confidence level and a desired precision of 4%, using the Wald method. When the research protocol was designed we expected to select at least 1776 individuals—one adult randomly selected in each household—in the three areas. However, when the project was approved, new data were obtained about the prevalence of some events of interest and about the population in each area, thus we increased the sample size and considered a proportional sample allocation to each urban unit. At the end a total sample with *n* = 1912 was obtained.

To select a random sample we needed the sampling frame, i.e., a complete list of all residents at least 18 years old who lived in each unit for at least 6 months. Given the lack of this type of sampling frame, we developed an alternative sampling frame based on the geographical coordinates of private households in each urban unit, combining GIS and statistical software. Non-classical households (hospitals, orphanages, military, etc.) and homeless were not included in this study. The urban planning team identified the geographical coordinates corresponding to households, providing the centroid of the polygons which is supposed to represent a building or a detached house. However, the spatial visualization shows roofs which may represent a household or a set of households, for example, a building with 7 floors with 2 households per floor. In the last case, we repeated the corresponding geographical coordinate 14 times. Field workers were needed to complete this exhaustive field work in order to provide a more realistic list of households in each area. This list was exported to SPSS statistical software and a random sample was generated for each area. The geographical coordinates of these samples were again exported to the ArcGIS program to mark them on a map to facilitate the work of the 14 interviewers trained to apply the UPHI-STAT questionnaire (described below).

The interviewers visited each random geographical coordinate, identifying how many households were eligible in each building and all adults living there. Each interviewer carried a set of cards to represent the number of households of a building and the number of adults of each household to randomly select one or more floor (if applicable) and one adult by household (if more than one adult was living there). If the selected adult was located at the moment and gave his/her written informed consent, the UPHI-STAT questionnaire was applied. Otherwise one or two more contacts were made to locate the selected adult before that household was considered as unreachable.

### UPHI-STAT Questionnaire—First Stage

In the scope of the project the UPHI-STAT questionnaire was developed and pre-tested (*n* = 49) by 14 local interviewers trained full-time for one week in November 2013 and three additional days at the end of January 2014. The survey was conducted in the three areas form January through end of March 2014. Several issues were explored in group discussions in the classroom and after field work with local communities in order to standardize procedures. As natives of Santiago island, the interviewers brought important contributions to the questionnaire and the data collection process. The questionnaire was written in Portuguese language—the official language in Cape Verde—but “crioulo” (Portuguese-based creole languages [32]) was also used by interviewers. Topics covered in this questionnaire include: 1) socio-demographic characterization; 2) perceptions and relationship with the living space; 3) selected household possessions and amenities; 4) self-reported health status and access to health services; 5) alcohol and tobacco use; 6) eating habits; 7) acquisition of and access to food; 8) physical activity; and 9) self-reported anthropometric data—weight, height and desired weight.

The final sample size was constituted by 145 participants in the formal unit, 1144 in the transition unit and 623 participants in the informal unit, in a total of 1912 respondents to UHPI-STAT Questionnaire.

### Anthropometric Measurement and Dietary Consumption—Second Stage

After completing the UPHI-STAT questionnaire, the respondent was invited to visit the team of 4 local nutritionists, in a near place within each unit, to collect anthropometric measures. Anthropometric measurements and body composition by bioelectric impedance were collected using a TANITA SC330 S^®^, accurate to 100*g*, minimum 2*kg* and maximum 270*kg*, calibration up to 300.000 uses. The measures obtained were: body weight, body fat, muscle mass, bone mass and body water. Participants’ height was assessed using a stadiometer Seca^®^, accurate to 1*mm*, where individuals were barefooted and with the weight evenly distributed on both feet, arms along the body, heels together, standing up straight and looking straight ahead. Waist and wrist circumferences were measured, according to standard procedures. Based on self-reported data and real measurements for weight and height, BMI was calculated according to Quételet equation and recoded into: underweight (BMI < 18.5), normal (18.5 ≤BMI< 25), overweight (25 ≤ BMI< 30), and obesity (BMI≥ 30*kg*/*m* ^2^), according to WHO recommendations [33]. The body fat (%) was categorized according to gender and age as described by previous works (e.g., [34, 35]).

Waist circumference (WC) was collected because it is used as a surrogate marker to define abdominal obesity. WC was measured midway between the lower rib margin and the iliac crest in the horizontal plane and it was recoded into categories to describe cardiometabolic risk by gender (Men: Increased risk—WC between 94 and 102 cm, Substantially increased risk—WC >102; Women: Increased risk—WC between 80 and 88 cm, Substantially increased risk—WC>88 cm) [36, 37]. The waist-to-height ratio (WHtR) was also obtained by dividing the WC value to the height of the individual. WHtR values >0.5 suggest cardiometabolic risk [37, 38].

Dietary consumption was assessed using a 24-hour diet recall applied through interview by the 4 local nutritionists. Through 24-hour diet recall, the nutritionist asked individuals to recall foods and beverages they consumed in the previous day to the interview (food types and amounts) from the moment they woke up in the morning until they went to sleep at night.

The second stage was completed by 599 of the 1912 participants (31.3%)—22 in the formal unit, 283 in the transition unit, and 294 in the informal unit. Ahead, some characteristics of the participants of the first and second stages will be explored.

### Pedometers—Third Stage

If the participant agreed, a pedometer was delivered with a registration form to collect the number of counted steps in a day (distinguishing working and non-working days), during a week. This stage was scheduled for a sample of about 200 inhabitants, according to the availability and durability of this type of equipment. The functioning of the pedometer was explained to the participants by the nutritionists. This stage was completed by a total of 118 participants: 1 in the formal unit, 80 in the transition unit and 37 in the informal area. In the statistical analysis the formal unit was not included.

### Qualitative Study—Fourth Stage

According to study design, based on a preliminary statistical data analysis, a qualitative study was developed to complement the quantitative approach, intending to add data and information for understanding how socio-economic-cultural inequalities and the urban morphology affect the health of residents. Ten focus groups with a total of 48 participants in the three units and two semi-structured interviews, with the directors of two non-governmental organizations in informal and transition units, were performed during November 2014. The focus groups of each unit were defined according to nutritional status, age group and gender of the participants. All of them had participated in the first two stages and some of them also participated in the third stage.

### Ethical Approval

The UPHI-STAT project was approved by two Ethic Committees—in Cape Verde (Comité Nacional de Ética em Pesquisa para a Saúde, Doc. n.52/2013) and in Portugal (Conselho de Ética of the IHMT, Doc. n.24-2013-PI), taking into account the field where the research takes place and the host and funding institutions of Portugal. Two copies of informed consent forms were presented to each participant, one to sign, that was given to the interviewer, and the other one to be kept by the participant.

### Statistical Analysis

Exploratory data analysis and classic descriptive statistics were used to describe and summarise the main variables of our datasets. Tables herein present *n*(%) for qualitative variables and median accompanied by interquartile interval (Quartile 1–Quartile 3), denoted by IQR, for non-normal quantitative variables. An assessment of the normality of the quantitative variables, using Kolmogorov-Smirnov and Shapiro-Wilk tests, was performed to ensure this prerequisite for many parametric tests. Another underlying assumption in parametric tests is the homogeneity of variances assessed by Levenne test. If assumptions of parametric test were not valid, we used non-parametric tests (Mann-Whitney-Wilcoxon and Kruskal-Wallis tests for independent samples or Wilcoxon test for paired samples). Associations between qualitative variables and comparisons of proportions were explored through Chi-Square test or the alternative Fisher exact test. Measures of association for dichotomous variables (e.g., *Φ* and Cramer’s V) were calculated when justified. Confidence intervals (95%CI) for proportions (e.g., prevalence of reports of physical activity) were obtained by Wilson and Agresti-Coull methods, preferable to the traditional Wald method [39]. To estimate the prevalence of overweight/obesity based on self-reported weight and height, we used the real measures to determine the sensitivity and specificity of the classification (overweight/obesity: yes or no), considering the measures performed by nutritionists as a gold standard. These concepts of diagnostic tests are introduced in the corresponding confidence intervals through the Blaker’s, Sterne, Clopper-Pearson and Wilson methods as described by Lang, Reiczigel, and co-authors [40, 41]. The concordance between nutritional status obtained by the categorization of BMI, based on self-reported measures and real measures, was assessed by Kappa coefficient. After these initial approaches, multivariate analyses were performed using generalized linear models, among others, for analysing multiple variables in an integrated way, adjusting for potential confounders. Some simple and multiple binary logistic regression models to obtain odds ratio (OR crude) and adjusted odds ratio (OR ajd) are also presented to identify possible factors associated to the adherence to nutritional status evaluation and also to explore the adherence to pedometers. The Hosmer and Lemeshow goodness of fit test and residual analysis were performed. SPSS version 22.0 (SPSS, Inc.), R [42] and EpiTools [43] programs were used to explore our datasets.

## Results

### Inhabitants of the urban units

Table 1 describes some characteristics of the residents within the three urban units, corresponding to the following neighbourhoods: Plateau (or Platô)– formal unit; a part of Palmarejo—transition unit and a part of Vila Nova—informal unit (see Fig 1). These units present differences in terms of urban planning and also exhibit marked disparities in terms of some socio-demographics variables of their inhabitants. There are no significant differences (*p* = 0.411) among urban units in terms of gender distribution of the respondents. In all units, there was a higher participation of women than men in the UPHI-STAT questionnaire. Significant differences at 5% level were found for all other variables presented in Table 1. The formal unit presents an older age structure (61.3% above 40 years old) and the transition unit a younger age structure (only 30.5% above 40 years old). Academic qualifications reveal a gradient in terms of the percentage of respondents not attending school—formal 3.5%; transition 5.4%; informal 14.2%—and attending secondary and high school, with similar values between formal and transition units (69.5% and 69.8%) and a lower percentage (48.5%) in the informal unit. Based on UPHI-STAT questionnaire, the unemployment rate is higher in the informal unit (28.7%), while in the other units is 6.2% (formal) and 17.5% (transition). The high percentage of retired people in the formal unit is expected, taking into account the age structure of this urban unit. Despite this, the percentage of students in this area (14.5%) is also higher than in the informal unit (9.3%). In terms of marital or family status and children, married and unmarried partners represent 31.1% and 34.0% in the formal and transition units, respectively, and about one quarter in the informal unit. However, the majority of the respondents have children, varying from 72.0% in the transition unit to 82.3% in informal unit. The median number of the children is higher in the informal unit, with three children, compared to two children in other units.

**Table 1:** Characteristics of the inhabitants of each neighbordhood/urban unit.

Variable	Formal	Transition	Informal	Total	p-value^a^
**Gender**, n (%)					0.411
Male	56 (38.6)	415 (36.3)	210 (33.7)	681 (35.6)	
Female	89 (61.4)	729 (63.7)	413 (66.3)	1231(64.4)	
**Age**, median (IQR)	47 (30–69)	33 (25–44)	37 (27–52.5)	35 (26–78.75)	<0.001^b^
**Age group**, n (%)					<0.001
18–25 years	20 (14.1)	289 (25.8)	125 (20.2)	434 (23,0)	
26–40 years	35 (24.6)	491 (43.8)	226 (36.5)	752 (39.9)	
41–65 years	44 (31.0)	305 (27.2)	205 (33.1)	554 (29.4)	
More than 65 years	43 (30.3)	37 (3.3)	64 (10.3)	144 (7.6)	
**Academic qualifications**, n (%)					<0.001
None	5 (3.5)	61 (5.4)	88 (14.2)	154 (8.1)	
Preschool	7 (5.0)	34 (3.0)	41 (6.6)	82 (4.3)	
Primary	31 (22.0)	248 (21.9)	191 (30.8)	470 (24.8)	
Secondary	59 (41.8)	418 (36.8)	245 (39.5)	722 (38.1)	
High school	39 (27.7)	374 (33.0)	56 (9.0)	469 (24.7)	
**Professional status**, n (%)					<0.001
Unemployed	9 (6.2)	200 (17.5)	179 (28.8)	388 (20.3)	
Worker	57 (39.3)	629 (55.1)	240 (38.6)	926 (48.5)	
Student	21 (14.5)	204 (17.9)	58 (9.3)	283 (14.8)	
Retired	44 (30.3)	53 (4.6)	51 (8.2)	148 (7.8)	
Other	14 (9.7)	56 (4.9)	94 (15.1)	164 (8.6)	
**Marital status**, n (%)					0.002
Without partner	100 (69.0)	748 (66.0)	462 (74.3)	1310 (68.9)	
With partner	45 (31.0)	386 (34.0)	160 (25.7)	591 (31.1)	
**Children**, n (%)					<0.001
No	30 (20.7)	320 (28.0)	110 (17.7)	460 (24.1)	
Yes	115 (79.3)	823 (72.0)	512 (82.3)	1450 (75.9)	
**Number of children**, median (IQR)	2 (1–4)	2 (1–4)	3 (2–5)	2 (1–4)	<0.001^c^
**Chronic disease**, n (%)					<0.001
No	110 (75.9)	904 (80.1)	425 (68.8)	1439 (76.1)	
Yes	35 (24.1)	224 (19.9)	193 (31.2)	452 (23.9)	
**Diet prescribed by doctor**, n (%)					0.041
No	134 (93.7)	1052 (92.0)	553 (88.8)	1739 (91.0)	
Yes	9 (6.3)	92 (8.0)	70 (11.2)	171 (9.0)	
**Weigh usually?**, n (%)					<0.001
No	72 (52.6)	549 (51.8)	384 (64.6)	1005 (56.1)	
Yes	65 (47.4)	511 (48.2)	210 (35.4)	786 (43.9)	

^a^Chi-Square test for qualitative variables

^b^Kruskal-Wallis test.

^c^Kruskal-Wallis test.

Briefly, some health-related variables were also analysed in each unit, revealing an excess of chronic conditions reported by inhabitants of the informal unit, compared with other units, despite the older age profile in the formal unit. Regarding chronic diseases, the most commonly reported was hypertension with 15.7%. Self-reported hypertension varied significantly among urban units (*p* < 0.001), with 19.3% in the formal unit, 11.4% in the transition unit and 22.5% in the informal unit. In the informal unit 11.2% of inhabitants reported that they were on a diet prescribed by a health professional, which was significantly different among urban units at 5% significance level (*p* = 0.041). Almost half of the residents of the formal and transition units reported the self-monitoring of their weight, compared to 35.4% in the informal unit.

### How the inhabitants see its neighbourhood—urban unit

Table 2 summarises participants’ opinions and perceptions regarding their neighbourhoods and/or city. In general, the inhabitants reported enjoy living in these three units, using a 5-point scale. In the informal unit, a slightly lower percentage of respondents reporting that the degree to which they liked living in its neighbourhood was “somewhat high” or “very” (the two top points of the scale), compared to respondents in the other two units (formal 82.7%; transition 80.3%; informal 76.2%). The most common reason given to live in the unit was tranquillity for respondents from Plateau and Palmarejo (formal 75.2%; transition 69.2%; informal 35.3%), whereas it was “housing and support from family” for respondents from the informal unit (formal 57.2%; transition 43.3%; informal 84.1%).

**Table 2:** Inhabitants’ perceptions of its neighbourdhood/urban unit *n*(%) and *p* − *value* associated to Chi-Square test.

Variables/Categories	Formal	Transition	Informal	Total	p-value
**Do you like to live here?**					0.007
Not at all	5 (3.4)	21 (1.9)	13 (2.1)	39 (2.1)	
Somewhat low	5 (3.4)	36 (3.2)	38 (6.2)	79 (4.2)	
Somewhat	15 (10.3)	165 (14.6)	94 (15.4)	274 (14.6)	
Somewhat high	46 (31.7)	406 (36.0)	235 (38.6)	687 (36.5)	
Very	74 (51.0)	500 (44.3)	229 (37.6)	803 (42.7)	
**Main reasons to live here**					
Tranquillity	109 (75.2)	792 (69.2)	220 (35.3)	1121 (58.6)	< 0.001
Housing and support from family	83 (57.2)	495 (43.3)	524 (84.1)	1102 (57.6)	< 0.001
Employment in the neighbourhood	30 (20.7)	156 (13.6)	32 (5.1)	218 (11.4)	< 0.001
Economic reasons	32 (22.1)	94 (8.2)	72 (11.6)	198 (10.4)	< 0.001
Other reasons	6 (4.1)	26 (2.3)	6 (1.0)	38 (2.0)	
**Evolution in the last 5 years**					< 0.001
Better	99 (70.2)	811 (77.7)	349 (60.1)	1259 (71.3)	
Equal	35 (24.8)	185 (17.7)	111 (19.1)	331 (18.7)	
Worse	7 (5.0)	48 (4.6)	121 (20.8)	176 (10.0)	
**What do you need to feel better here?**					
Increased security	90 (62.1)	760 (66.4)	475 (76.2)	1325 (69.3)	< 0.001
Health Center	30 (20.7)	717 (62.7)	430 (69.0)	1177 (61.6)	< 0.001
Cultural and recreational activities	60 (41.4)	518 (45.3)	307 (49.3)	885 (46.3)	0.128
Global environment	59 (40.7)	448 (39.2)	326 (52.3)	833 (43.6)	< 0.001
Garbage removal in public spaces	57 (39.3)	477 (41.7)	241 (38.7)	775 (40.5)	0.446
Centres for elderly people	67 (46.2)	351 (30.7)	326 (52.3)	744 (38.9)	< 0.001
Sport facilities	48 (33.1)	479 (41.9)	198 (31.8)	725 (37.9)	< 0.001
Gardens and green spaces	27 (18.6)	443 (38.7)	126 (20.2)	596 (31.2)	< 0.001
Public spaces	25 (17.2)	296 (25.9)	124 (19.9)	445 (23.3)	0.004
Public transportation	29 (20.0)	180 (15.7)	115 (18.5)	324 (16.9)	0.205
Improvement of the accessibility	28 (19.3)	162 (14.2)	126 (20.2)	316 (16.5)	0.003
Schools and kindergartens	8 (5.5)	160 (14.0)	118 (18.9)	286 (15.0)	< 0.001
More stores and shops	4 (2.8)	86 (7.5)	48 (7.7)	138 (7.2)	0.096
More coffee shops	5 (3.4)	34 (3.0)	60 (9.6)	99 (5.2)	< 0.001
Other	6 (4.1)	86 (7.5)	37 (5.9)	129 (6.7)	0.193

Employment was reported as a reason to live in the neighbourhood, in an expected way, decreasing from formal (20.7%) to informal (5.1%). In the last 5 years, the evolution of the three urban units was favorably classified. Very few respondents (≤5.0%) pointed out a worse situation in the formal and transition units. However, in the informal unit, 20.8% reported a negative evolution of the unit.

When we asked what is necessary to improve the neighbourhood, insecurity appears as the main concern in the three units, notwithstanding with significant differences (*p* < 0.001) among units, with more than three-quarters (76.6%) of the respondents of informal unit referring the need for more security. Focus group discussions also reinforce this pattern in all units. Need for improvement in the global environment was reported by respondents of the units significantly differently (*p* < 0.001) and with an increasing trend from formal to informal units. The lack of some facilities was also reported, namely the need for a health centre, centres for the elderly and sport facilities, with significant differences among the urban units (*p* < 0.001, in all cases). The need for a health centre was reported by 62.7% and 69.0% in transition and informal units, respectively, but only by 20.7% in the formal unit, where there is a hospital. Regarding the need for centres for the elderly, perhaps the age structure and family explain the “V” pattern, with a lower value in the transition unit (30.7%) and higher values in other units—formal 46.2% and informal 52.3%. This type of pattern appears also for improvement of accessibility, although the issue was reported less frequently. On the other hand, an inverted “V” pattern with a higher value in the transition unit was observed in relation to the need for sport facilities, gardens and green space and public spaces.

There are not significant differences among urban units regarding the need for entertainment and cultural activities (referred by 46.3% of the respondents), garbage removal in public spaces (40.5%), public transportation (16.9%) and more stores and shops (7.2%). Although the city does not have public transportation, this fact was not among the most mentioned issues, perhaps, due to the network of taxis and private buses easily accessible at low prices. Schools and kindergartens were mentioned by 15% of the respondents, again with an increased trend from formal to informal unit.

### Adherence to nutritional status evaluation and the use of pedometers

As mentioned before, only 599 of the 1912 participants (31.3%) completed the second stage of our study: going to the places where the nutritionists collected the anthropometric measures and food intake information, through a structured 24 Hour Recall questionnaire. By convenience, within this text, we use a short description to designate this stage: “adherence to nutritional status evaluation”. It is important to understand what characteristics of the participants (e.g., age, sex, education, occupation) or family-related variables (marital status), and environment contexts (local where he/she lives) that may influence adherence to this stage. Considering adherence to nutritional status as a binary dependent variable (yes: 599 cases; no: 1313 cases), several simple logistic regression models were initially performed to identify a set of variables with *p* ≤ 0.20. To avoid confounding, these variables and other variables (with a particular meaning) were analysed simultaneously using multiple logistic regression models to identify possible factors that could explain adherence to nutritional status evaluation. Some results are presented in Table 3 for a sub-sample without missing values of size *n* = 1833. After adjustment for potential confounders, the urban unit was still one of the significant variables, revealing a higher level of adherence of residents of the transition and informal units compared to formal unit. According to the fitted model, individuals from informal unit were nearly six times (95%CI [3.035, 12.287]) more likely to participate in this stage of our study, compared with the formal unit. Women were also revealed to be more likely to participate in this stage than men. Participants who were unemployed, students and other workers (this category includes the housekeepers), compared to participants who reported to be working, more frequently visited the team of nutritionists. The simple logistic regression model and some multiple regression models suggested a higher participation in nutritional status evaluation by participants who self-reported suffering from chronic conditions. However, this significant association disappears when BMI based on self-reported measures of height and weight was included in the models. When included indirectly self-reported BMI in the model (*n* = 1342), it was found that a higher BMI was associated with the adherence to nutritional status evaluation. This situation is particularly relevant to the next subsection.

**Table 3:** Adherence to nutritional status evaluation—significance and odds-ratios with the 95% confidence intervals (95%C.I.), obtained by simple logistic (OR crude) and multiple regression (OR adj) models.

Variable/Categories	p-value	OR crude	95% C.I.	p-value	OR ajd	95% C.I.
**Urban unit**						
Formal ^(a)^	<0.001			<0.001		
Transition	0.012	1.838	1.145–2.950	0.006	2.624	1.325–5.196
Informal	<0.001	4.996	3.091–8.076	<0.001	6.107	3.035–12.287
**Gender**						
Male ^(a)^						
Female	<0.001	2.057	1.658–2.552	<0.001	1.715	1.285–2.290
**Age group**						
18–25 yr ^(a)^	0.002			0.209		
26–40 yr	0.312	0.875	0.675–1.134	0.201	0.772	0.520–1.148
41–65 yr	0.019	1.375	1.053–1.797	0.87	1.041	0.644–1.683
> 65 yr	0.335	1.217	0.816–1.814	0.541	0.760	0.315–1.834
**Education**						
None ^(a)^	<0.001			0.287		
Preschool	0.756	0.925	0.563–1.517	0.934	1.034	0.473–2.260
Primary	0.050	0.689	0.475–0.999	0.225	1.456	0.793–2.685
Secondary	<0.001	0.489	0.343–0.698	0.881	1.049	0.562–1.957
High School	<0.001	0.350	0.239–0.514	0.931	0.971	0.502–1.877
**Professional status**						
Worker ^(a)^	<0.001			0.002		
Unemployed	<0.001	2.067	1.603–2.664	0.009	1.602	1.124–2.283
Student	0.007	1.496	1.117–2.005	<0.001	2.270	1.466–3.514
Retired	0.010	1.637	1.128–2.377	0.319	1.407	0.719–2.751
Other	<0.001	3.538	2.514–4.980	0.041	1.703	1.023–2.835
**Marital status**						
Without partner ^(a)^						
With partner	0.201	0.871	0.705–1.076	0.567	1.088	0.815–1.454
**Children**						
No ^(a)^						
Yes	0.158	1.18	0.938–1.18	0.354	1.196	0.819–1.746
**Chronic condition**						
No ^(a)^						
Yes	<0.001	1.905	1.530–2.372	0.147	1.284	0.916–1.800
**Diet prescribed**						
No ^(a)^						
Yes	0.005	1.590	1.153–2.192	0.807	1.057	0.675–1.656
**Self-reported BMI**	<0.001	1.065	1.036–1.094	0.001	1.056	1.023–1.090

^(a)^ Reference category for qualitative variables

Taking into account the lack of published works about the use of pedometers in Cape Verdean communities, it is important to explore the adherence to this type of tool. Only one individual delivered the report sheet of steps obtained with the pedometer in the formal unit. In this stage, the inhabitants of the transition unit accepted and brought back the sheet of steps more frequently than informal unit. Using a similar approach, with logistic regression models, the most important associated variables were urban unit (*p* = 0.002), education, considering as the reference category “none or preschool”, the adjusted Odds Ratios were 6.96, *p* = 0.001 for primary; 7.70, *p* < 0.001 for secondary and 8.81, *p* < 0.001 for high school. In some models diet prescribed by health professional (*p* = 0.034) was also a significant variable at the 5% level.

### Overweight/obesity classifications and abdominal adiposity indicators with focus on women

Considering the two BMI measures (indirectly self-assessed BMI based on self-reported weight and height by participants *vs* real BMI collected by the nutritionists using bioelectric impedance), two different values of prevalence of the overweight/obesity, defined by BMI ≥25*kg*/*m* ^2^ appear. However, there are some issues that we need to address. As the adherence to nutritional status evaluation is conditioned by self-reported BMI, this result leads to the conclusion that anthropometric measures obtained by bioelectric impedance in the subgroup (*n* = 599) should be higher than the remaining group (*n* = 1313), who had self-reported measures. Consequently, using the real BMI to quantify the overweight/obesity will result in an overestimation of the corresponding prevalence in the studied population. On the other hand, the estimation of prevalence of overweight/obesity will be underestimated by self-reported measures. For a sub-sample of size *n* = 395 without missing values in any variable involved, the concordance between these two BMI was obtained, revealing a significant agreement (*Kappa* = 0.601, *p* < 0.001). Despite this agreement, 38.2% (13/34) of the participants indirectly self-classified as underweight were classified by nutritionists as normal BMI. On the other extreme, 30.6% (38/124) of the participants indirectly self-classified as normal BMI were classified as overweight, and 31.0% (27/87) of the respondents self-classified as overweight BMI were obese. Based on the self-reported measures for 1399 individuals, 553 (39.5%) were reported as overweight/obesity with a 95%CI [37.0, 42.1], obtained by Wilson method. In the same way, after excluding four pregnant women, the magnitude of the same event was 338/595 (56.7%) 95%CI [52.7, 60.8], using the real BMI. Taking into account the issues associated with both confidence intervals, we use concepts of an imperfect diagnostic test (self-reported) to correct the first estimate. In fact, it is possible to use the sub-sample with both BMI (*n* = 395) to estimate the sensitivity (80.6%) and the specificity (90.2%) of the indirect binary self-classification as overweight/obesity (yes, no), considering the measures performed by nutritionists as a gold standard. After that, confidence limits for prevalence of overweight/obesity adjusted for sensitivity and specificity are calculated—95%CI [38.4, 45.7]—using Blaker’s, Sterne, Clopper-Pearson and Wilson methods as described by [40, 41].

For each urban unit, the differences between women and men were frequently significant, as expected, at least for some measures. In this work, we do not present this type of results. Deliberately, we give a particular attention to results for women, because women present higher prevalence of overweight/obesity, and on the other hand, they show a higher discrepancy between the actual weight and the desired weight. In addition, women participated more than men in all stages of our study, which is a good indication for future health promotions in these urban units. Table 4 summarises results for measures that were self-reported, those measured by nutritionists, and others derived from these for women by urban unit. Before the table analysis, initially analysing in more detail the paired subgroup of women simultaneously with both values, there was a significant difference by Wilcoxon signed rank test (*n* = 358; *p* < 0.001). It was noticed that women tend to report a height greater than the real value. In terms of weight, there is no significant difference between self-reported and measured weight (*n* = 327; *p* = 0.879). Thus, in this particular context, the problem with indirectly self-assessment BMI is caused by overestimation of height.

**Table 4:** BMI and cardiometabolic risk for women according to urban unit.

Only women	n	Formal	n	Transition	n	Informal	n	Total	p-value
**Self reported** (SR)^a^									
**SR weight** (Kg)	70	66.0 (60.0–74.0)	609	64.0 (57.0–72.0)	275	67.0 (58.0–76.0)	954	65.0 (57.0–74.0)	0.006
**SR height** (m)	75	1.62 (1.57–1.68)	647	1.63 (1.59–1.69)	324	1.61 (1.57–1.65)	1046	1.63 (1.58–1.68)	< 0.001
**SR BMI** (kg/m2)	62	24.4 (22.0–27.3)	570	23.9 (21.2–26.7)	234	25.7 (22.0–29.1)	866	24.3 (21.5–28.0)	< 0.001
**BMI classification** ^b^									< 0.001
Underweight	4	6.5	42	7.4	15	6.4	61	7.0	
Normal weight	31	50.0	321	56.3	89	38.0	441	50.9	
Overweight and obese	27	43.5	207	36.3	130	55.6	364	42.0	
**Measured**									
**Weight** (Kg)	16	71.9 (56.0–85.3)	225	66.8 (56.0–78.4)	206	66.7 (58.5–78.5)	447	66.9 (21.5–27.6)	0.516
**Height** (m)	16	1.59 (1.54–1.60)	224	1.60 (1.55- 1.65)	207	1.59 (1.55–1.63)	447	1.59 (1.55–1.64)	0.181
**BMI** (kg/m2)	16	28.7 (22.1–32.8)	223	26.1(22.0–30.2)	206	26.8 (23.4–31.4)	446	26.5 (22.6–30.9)	0.215
**BMI classification**									0.435
Underweight	0	0.0	20	8.9	14	6.8	34	7.6	
Normal weight	5	31.3	71	31.7	55	13.3	131	29.4	
Overweight and obese	11	68.8	133	59.4	137	66.5	281	63.0	
**Fat mass** (%),	16	35.3 (29.2–42.1)	223	33.9 (24.8–39.1)	204	35.8 (30.2–41.4)	443	34.9 (27.2–39.9)	0.005
**Fat mass classification**									0.036
Underfat	0	0.0	10	4.5	4	2.0	14	3.2	
Healthy	2	12.5	46	20.6	33	16.2	81	18.3	
Overfat	3	18.8	30	13.5	13	6.4	46	10.4	
Obese	11	68.8	137	61.4	154	75.5	302	68.2	
**WC (cm)**	15	93.5 (79.0–98.0)	221	90.0 (78.0–99.0)	206	93.3 (81.0–103.0)	442	91.0 (79.0–101.0)	0.046
**WC risk—WHO** ^c^									0.468
None	4	26.7	64	29.0	44	21.4	112	25.3	
Increased	3	20.0	33	14.9	33	16.0	69	15.6	
Highly increased	8	53.3	124	56.1	129	62.6	261	59.0	
WHtR (cm)	15	0.60 (0.50–0.62)	220	0.56 (0.48–0.62)	205	0.59 (0.52–0.65)	440	0.57 (0.50–0.63)	0.017
**WHtR risk** ^d^									0.074
≤0.5	4	26.7	69	32.4	45	22.4	118	27.5	
>0.5	11	73.3	144	67.6	156	77.6	311	72.5	

^a^For these set of quantitative variables and other, *n*, median and IQR are represented in parenthesise and p-value corresponds to Kruskal-Wallis test.

^b^For qualitative variables, *n* and percentage are presented and p-value corresponds to Chi-Square test

^c^Cardiometabolic risk according to waist circumference WHO

^d^Cardiometabolic risk according to WHtR

Table 4 shows the self-reported weight, height, and BMI, where we found significant differences among urban units, indicating an unfavorable situation for the informal unit. In percentage terms, the BMI classification indicates 55.6% of overweight and obesity in the informal unit, despite having a younger age structure than the formal unit. Also due to the reduction in sample sizes, using the measures obtained by nutritionists, particularly in the formal unit, there were no significant differences across urban units. Even taking into account overestimation of the percentage of overweight/obesity it is clear that the three units present high levels. Using fat mass (%), the Kruskall-Wallis test reveals significant differences among units (*p* = 0.005), with similar percentiles for informal and formal units and a better situation in transition unit. The fat mass classification presented significant differences only at 5% level (*p* = 0.036), and aggregating overfat and obese categories, we found 87.6%, 74.9% and 81.9% in formal, transition and informal units, respectively, for women observed by nutritionists. These percentages are higher than the ones corresponding to overweight and obese categories based on BMI. Abdominal adiposity indicators also show a critical situation, according to the recommendation of the WHO, with the percentage of women with metabolic risk (increased and highly increased) about 74.6%, without significant differences among urban units. According to WHtR, 77.6% presented metabolic risk. In general, Table 4 shows a better situation in the transition unit and a worse situation for women of the informal unit. For some indicators, the informal unit presents values similar to the formal unit which is characterized by an older age structure.

### Physical activity by gender and urban unit

Table 5 describes results relating to physical activity in global terms, and in work and in leisure time, for women and men in the three urban units. Concerning physical activity for women, the overall percentage was 67.4% (95%CI [64.8,70.0]), with significant differences among urban units only at the 5% significance level (*p* = 0.025). In the transition unit, about 70.4% reported global physical activity. Physical activity in men was higher, around 85.2% (95%CI [82.3, 87.6]), without significant differences among urban units (*p* = 0.266). Women in the formal unit reported more physical activity at work, however, the percentage was only 16.3% (*p* = 0.002). Women in the informal unit reported less physical activity in leisure time *p* = 0.005). Among men, there is a significant difference among units (*p* < 0.001), and it is the informal unit that presents more physical activity at work. In terms of physical activity of men at leisure no significant differences were found between units. In terms of intensity, both men and women tended to report moderate physical activity. Regarding physical activity in leisure time, the magnitude of the practice was very discrepant by gender, with a 95%CIs [22.6, 27.4] for women and [53.2, 60.2] for men.

**Table 5:** Physical activity—global, work, and leisure—by gender and urban unit.

**WOMEN**	Formal (n = 89)	Transition (n = 729)	Informal (n = 413)	Total (n = 1231)	p-value
**Physical activity—global**, n (%)					0.025
No	35 (39.3)	216 (29.6)	150 (36.3)	401 (32.6)	
Yes	54 (60.7)	513 (70.4)	263 (63.7)	830 (67.4)	
**Physical activity—work**, n (%)					0.002
No	72 (83.7)	685 (94.0)	377 (91.3)	1134 (92.3)	
Yes	14 (16.3)	44 (6.0)	36 (8.7)	94 (7.7)	
**If yes…**					
Moderate	6 (42.9)	30 (68.2)	21 (58.3)	57 (60.6)	0.514
Intense	4 (28.6)	8 (18.2)	9 (25.0)	21 (22.3)	
Moderate and intense	4 (28.6)	6 (13.6)	6 (16.7)	16 (17.0)	
**Physical activity—leisure**, n (%)					0.005
No	62 (70.5)	528 (72.4)	333 (80.6)	923 (75.0)	
Yes	26 (29.5)	201 (27.6)	80 (19.4)	307 (25.0)	
**If yes…**					
Moderate	10 (38.5)	129 (64.2)	52 (65.0)	191 (62.2)	0.025
Intense	8 (30.8)	51 (25.4)	20 (25.0)	79 (25.7)	
Moderate and intense	8 (30.8)	21 (10.4)	8 (10.0)	37 (12.1)	
**MEM**					
**Physical activity—global**, n (%)					0.266
No	12 (21.4)	56 (13.5)	33 (15.7)	101 (14.8)	
Yes	44 (78.6)	359 (86.5)	177 (84.3)	580 (85.2)	
**Physical activity—work**, n (%)					<0.001
No	45 (83.3)	346 (83.6)	139 (66.2)	530 (78.2)	
Yes	9 (19.7)	68 (16.4)	71 (33.8)	148 (21.8)	
**If yes…**					
Moderate	5 (55.6)	27 (39.7)	29 (40.8)	61 (41.2)	0.276
Intense	1 (11.1)	28 (41.2)	21 (29.6)	50 (33.8)	
Moderate and intense	3 (33.3)	13 (19.1)	21 (29.6)	37 (25.0)	
**Physical activity—leisure**, n (%)					0.242
No	23 (41.8)	169 (40.8)	100 (47.8)	292 (43.1)	
Yes	32 (58.2)	245 (59.2)	109 (52.2)	386 (56.9)	
**If yes…**					
Moderate	5 (15.6)	76 (31.0)	33 (30.3)	114 (29.5)	0.361
Intense	15 (46.9)	107 (43.7)	44 (40.4)	166 (43.0)	
Moderate and intense	12 (37.5)	62 (25.3)	32 (29.4)	106 (27.5)	

As global physical activity also includes walking, it is important to refer the complementary information of the third stage, based on the report sheets of steps obtained with the pedometer, available only for transition and informal unit (Table 6). We analysed the number of days—total, working and non-working—registered, as well as the number of steps on each one. The sample presented a median (interquartile range) of total steps/day of 5234.8 (3458.2 − 7227.0), with no significant differences between the two units, even though the transition unit presented a higher median. As for the number of days with registry, the informal unit had a lower median of working days and a higher median of non-working days, comparing with the transition unit (*p* < 0.050 and *p* < 0.010, respectively). By gender, men walked significantly more than women (*p* < 0.001), with a difference in the medians of approximately 2000 steps/day.

**Table 6:** Number of steps registered in working and non-working days in the transition and informal units and by gender.

**By Urban unit:**	**Transition**	**Informal**	Total	p-value
Days registered (Total)^a^	7.0 (7.0–8.0)	8.0 (7.0–8.0)	7.0 (7.0–8.0)	0.291
Steps/day (Total)	5464.4 (3673.0–7222.2)	4443.0 (3274.6–6778.6)	5234.8 (3458.2–7227.0)	0.322
Total: < 5000steps/day, n (%)^b^	32 (40.0)	22 (59.5)	54 (45.8)	0.022
Working days	5.0 (3.5–6.0)	4.0 (0.0–6.0)	5.0 (2.0–6.0)	0.040
Steps/working day	5827.2 (3742.6–8144.6)	5549.4 (2755.3–7248.7)	5863.4 (3418.7–8144.6)	0.477
Working days: < 5000 steps/, n (%)	26 (39.4)	11 (45.8)	37 (40.7)	0.063
Non-working days	2.0 (1.0–3.0)	3.0 (2.0–7.0)	2.0 (1.0–4.0)	0.002
Steps/non-working day	4562.1 (2639.0–7820.0)	3969.6 (2364.0–8105.6)	4295.4 (2585.5–7820.0)	1.000
Non-working days: < 5000 steps, n (%)	36 (54.5)	23 (63.9)	59 (57.8)	0.542
**By gender:**	**Male**	**Female**		
Days registered (Total)	8.0 (7.0- 8.0)	7.0 (7.0- 8.0)	7.0 (7.0- 8.0)	0.412
Steps/day (Total)	6778.6 (5281.4–9452.1)	4831.3 (3270.0–6765.8)	5234.8 (3458.2–7227.0)	0.004
Total: < 5000 steps/day, n (%)	5 (18.5)	49 (54.4)	54 (45.8)	0.009
Working days	5.0 (2.0–6.0)	5.0 (1.0–6.0)	5.0 (2.0–6.0)	0.707
Steps/working day	7037.2 (5887.0–9104.8)	5179.6 (3087.8–7194.0)	5863.4 (3418.7–8144.6)	0.036
Working days: < 5000 steps, n (%)	4 (18.2)	33 (48.5)	37 (40.7)	0.020
Non-working days	2.0 (2.0–5.0)	2.0 (1.0–4.0)	2.0 (1.0–4.0)	0.315
Steps/non-working day	6696.5 (3281.5–10147.0)	4065.3 (2575.5–7091.1)	4295.4 (2585.5–7820.0)	0.051
Non-working days: < 5000 steps, n (%)	9 (36.0)	50 (64.9)	59 (57.8)	0.044

^a^For quantitative variables, *n*, median and IQR are represented in parenthesise and p-value corresponds to Mann-Whitney test.

^b^For qualitative variables, *n* and % are presented and p-value corresponds to Chi-Square test.

### Relationship between physical activity in leisure time and the perception of security and lack of infrastructures

Possible relationships between physical activity in leisure time and (i) the need for increased security, and (ii) lack of some infrastructures were explored using some binary variables already described in Table 2. Stratifying by urban unit and/or gender, we found some significant associations. Among all participants in the informal unit, physical activity in leisure time presented a weak negative association (Φ = −0.082, *p* = 0.040) with perception of need for more security (i.e., participants who reported insecurity, or the need for more security, seem to be less active in leisure time). Overall and among men in the transition unit, there was a positive association between physical activity in leisure time and the need for more sport facilities (Φ = 0.199, *p* < 0.001 and Φ = 0.109, *p* < 0.001), respectively). There was a positive association between physical activity in leisure time and the need for more gardens and green spaces for men and overall (Φ = 0.199, *p* < 0.001 and Φ = 0.109, *p* < 0.001, respectively) in transition unit and also for men of the formal unit (Φ = 0.350, *p* = 0.009)). Men who engage in physical activity during leisure time seem to require more infrastructures in their neighbourhood.

Exploring this type of association with another binary variable “walking and cycling” (responses to the question *Do you walk or use a bicycle (pedal cycle) for at least 10 minutes continuously to get to and from places?*) shows a similar trend. Stratifying by gender, a negative association between “walking and cycling” and the need for more security appears for women (Φ = −0.094, *p* < 0.001). Stratifying by gender and urban unit, the significance of this association was verified for women of the transition and informal units (Φ = −0.111, *p* = 0.003 and Φ = −0.102, *p* = 0.038, respectively). The relationship between “walking and cycling” and “gardens and green spaces” presented significant positive associations (results omitted) for both men and women of the transition and informal units. Again, participants who walk and bike tend to report a need for more gardens and green spaces.

Observation during fieldwork revealed unequal distribution of infrastructures for physical activity within the city. Focus groups allowed us to understand some determinants of physical activity, according to the perceptions of the populations of the three areas under study. Discussions of the focus groups seem to reinforce that insecurity of the city is a potential explanatory factor for not practicing physical activity, particularly in the informal unit, as suggested by some participant opinions:

“*Also in our neighbourhood, where we are, due to the level of insecurity, people do not feel safe to go out and walk*.” (Focus Group Participant, Men, 37 years)

“*I’ve been the victim of robbery. I was walking*.” (Focus Group Participant, Men, 43 Years)

“*It is late for me and there is no security on the street …” “There’s a lot of insecurity in the neighbourhood, I’d rather not go to practice physical exercise … Yes this is my problem and here where I live there are many delinquents*.” (Focus Group Participant, Women, 28 years)

“*No one can leave the house very early, you never know who you’ll meet on the walk, where you can be assaulted, be beaten*.” (Focus Group Participant, Women, 53 years)

## Discussion

In addition to the differences in terms of urbanization of the three units, Table 1 shows how the inhabitants differed across urban units for some variables. With the exception of gender distribution, we found statistically significant differences in terms of socioeconomic variables (e.g., age, academic qualifications and professional status), family-related variables (marital status and number of children) and also in terms of some health-related variables (chronic diseases, namely, self-reported hypertension, diet prescribed by a health professional and self-monitoring of the weight). In the informal unit, inhabitants presented an unfavorable situation.

Overall, self-reported hypertension percentage was 15.7%, varying significantly among urban units, with 19.3% in the formal unit, 11.4% in the transition unit and 22.5% in the informal unit. This overall percentage was similar to the percentage of 14.5% found in Cape Verde, in 2007, for adults between 25–64 (*n* = 2200), but is very dissimilar to the percentage—34.9%—found when hypertension was measured using conventional procedures [44, 45].

Doulougou et al [46] studied the prevalence of hypertension in formal and informal areas of the city of Ouagadougou in Burkina Faso and found a global prevalence of hypertension of 18.6%. Although without significant difference between urban areas, a higher percentage was found in the formal area (21.4%) compared to the informal area (15.3%) which had a younger age structure. In our study, although formal unit presents an older age structure than the informal unit, even so participants of the informal unit reported percentages of chronic diseases higher than in the formal unit (31.2% *vs* 24.1%). Doulougou et al [46] found lower values compared to our study, without differences between informal and formal areas (17.1% *vs* 17.2%).

Our study concerns a very small scale, where the three urban units are geographically near each other. Despite this proximity, some related aspects of the nutritional status of participants varied significantly between urban units. Gender disparities in overweight and obesity are also marked in Cape Verde. This fact is described for some developing countries, particularly in the North Africa and Middle East, where the cultural environment favors a larger body size because is linked to fertility, prosperity and healthfulness in women [47]. In fact, in Africa it seems that obesity is more prevalent in middle-aged women in urban areas [48–50]. These results are consistent with other studies in populations of African countries [51, 52].

Women of the three urban units present significant differences in terms of BMI calculated from self-reported measures, fat mass, waist circumference and waist-to-height ratio (Table 4). The percentage of overweight and obesity based on self-reported measures differed across urban units, despite no significant differences found in the smaller samples with measures collected by nutritionists. It is possible that the reduction of the sample size from first to second stages, the small sample size of the formal unit, and the influence of the BMI in the participation in the second stage may explain theses findings.

In a national study [44, 45], based on self-reported weight and height, the percentages of overweight was 36.9% and obesity was 10.5%. Also in Cape Verde, Ng et al [53] reported percentages of overweight around 44.0% [41.3, 47.0] and obesity of 15.4% [13.9,17.1], for women more than 20 years old. For men, these percentages were 31.8% [29.4,34.3] and 7.0% [6.2,7.8], respectively. Our study indicated 42% of overweight and obesity among women (see Total column in Table 4) and among men 35.5% (data not shown). Our value is lower than the previous one, particularly for women. However, we found very discrepant values between transition (36.3%) and informal unit (55.6%). In Ouagadougou (Burkina Faso), Doulougou et al [46] described lower percentages of overweight and obesity overall, with a higher percentage of overweight and obesity in the formal area (28.4%) than in the informal area (17.6%). Discrepancies between urban and rural areas and also across urban areas within a country or different countries are pointed out by several authors (e.g., [3, 53]). Thus, local communities would benefit from local studies that show particularities of a city at a micro-level scale.

No significant differences were found among urban units, in terms of cardiometabolic risk of women, according to WHO or WHtR definitions, with 74.6% and 72.5%, respectively. For men, it should be noted that these percentages were lower 22% and 35.9% (data not shown). In Tunisia, Ati et al [37] reported higher percentages, based on WHtR, for women (82.4%) and men (69.6%) between 35 and 70 years old. Ware et al [38] found lower values for women in a study with participants from rural and urban areas of South Africa in terms of WHtR and waist circumference.

Within the multifactorial nature and the complexity of health problems, neighbourhood environment factors such as safety from crime and traffic were associated with overweight among Nigerian adults [54]. Among others, Sallis et al [13] describe the role of built environments in physical activity, and indirectly in obesity and cardiovascular diseases. In some developing countries under nutrition transition, a greater impact on the physical activity of women has been highlighted [47]. Doulougou et al [46] found an association between physical inactivity and hypertension in both formal and informal areas. In our study, for women, overall physical activity was 67.4% (95%CI [64.8,70.0]), with differences among urban units (*p* = 0.025). For men, it was 85.2% (CI95% [82.3,87.6]), without significant differences among urban units. The difference between the percentages of women and men who reported physical activity in leisure time was particularly discrepant (women: 95%CI [22.6, 27.4] *vs* men: [53.2, 60.2]). Pedometers also indicated men walked significantly more than women (*p* < 0.001), with a difference of approximately 2000 steps/day. In this context, recent studies show the link between pedometers and health and promotion health messages, but there is a lack of this type of data from developing countries, specifically African countries [55]. The few studies are focused on sub-groups of population (e.g., adolescents, women, rural activities) or associated to intervention studies, with small sample sizes.

Walking has been suggested as a good from of physical activity in particular in developing countries, because is inexpensive and accessible for a vast population (see [56] and references therein). However, particularly in the cities, there are many aspects to be considered to promote walking, with local urban planning and security being two of them. A borderline association was found between the perception of more security and physical activity in leisure time in the informal unit. Study participants who reported insecurity tend to be inactive in leisure time. Insecurity was also negatively associated with walking and cycling for women in transition and informal units. The qualitative study also reveals this concern, with some women justifying their lack of physical activity (including walking) by citing insecurity of the environment and absence of adequate infrastructures. Participants who practiced physical activity in leisure time or walking and cycling tended to report the need for more infrastructures (sport facilities, gardens and green spaces) in their neighbourhoods. Young men of the transition unit with higher academic qualifications revealed more involvement in physical activity in leisure time. On the other hand, older women of the informal unit who perceived insecurity tended to be inactive.

Cape Verde is described by Zoettl [57] as the country of “morabeza” (gentleness). However, it has been facing problems of youth delinquency and gang-related violence, in the last decade. In 2007, a report conducted by United Nations and Cape Verde Ministry of Justice [58] highlighted that the most of the crimes experienced by the population did not necessarily involve violence. The reporting rates were much lower than in other African countries and among the lowest in the world, with the exception of sexual offences. Nevertheless, the crime level in Praia was higher than in other two African capitals—Gaborone (Botswana) and Maseru (Lesotho). Both citizens and police respondents ranked unemployment and drug consumption as the main causes of crime [58].

In our study, the highest unemployment rate (28.8%) was found in informal unit (Table 1). The participants reported to like very much living in these neighbourhoods and/or city and they had positive views regarding the evolution of the neighbourhoods in the last 5 years. However, the percentage of the inhabitants expressing this positive evaluation was the lowest in the informal unit. The need for more security in the neighbourhood and/or city appeared as the main concern of the participants of the three urban units, notwithstanding with significant differences among units, with a higher percentage (76.6%) among respondents of the informal unit. Several studies, for example, an American study by McGinn et al [59], showed that both real and perceived crime levels in neighbourhoods discouraged physical activity of their residents. Oyeyemi et al [54] found that in Nigeria, consist with some previous studies, a neighbourhood with crime at night may have negative influence on physical activity. The fear of crime can impact physical activity and decrease confidence and remove the desire to go outdoors. In the urban area of Pelotas, Brasil, Mendes et al [60] reported lower percentage of the physical activity in leisure time and transportation (10.5% and 51.7%) in groups with high levels of insecurity. However, the association between physical activity and perceived insecurity was not significant. As reinforced by Kjellstrom and Mercado [1], violence and crime in poor urban areas have serious implications for trust and well-being. In the State of African Cities Report [61], women (and children) of urban poor areas are stated to be most at risk from disease and other social and environment problems.

### Strengths and Limitations

In the UPHI-STAT research project we developed a sampling frame based on the geographical coordinates of private households in each area, combining GIS and statistical software. This strategy may be applicable to similar settings, where the identification of geographical coordinates of households is possible. A stratified random sample of size *n* = 1912 was obtained using proportional allocation. In each household, one adult was selected at random, from the list enumerated of all adults (at least 18 years old and having lived for at least six months in the respective urban unit). Each participant was interviewed in a first contact or using two more additional contacts, in case of failure of the first or second contacts. After the UPHI-STAT questionnaire was administered, the selected adult was invited to be observed by a nutritionist in a nearby location, to collect data about dietary consumption, anthropometric measurements and body composition by bioelectric impedance. In the third stage, walking was measured via pedometers. In Cape Verde, to our knowledge, there were no previous studies using pedometers. The use of appropriate equipment is particulary relevant in the African context.

Many studies are focused on self-reported and measured BMI and do not explore other important body composition measures. It is well-known that BMI does not differentiate between fat mass and fat-free mass, being a poor predictor of body fat [62]. Among several methods, bioelectrical impedance analysis is previously described [62] as safe, non-invasive, quick, and relatively inexpensive. However, in an African setting the last characteristic is not always valid. All equipment of the UPHI-STAT project was bought in Portugal due to their unavailability in Cape Verde. Calibration was performed in Portugal and some variations may have occurred during air transportation, although all measures were considered to avoid posterior variation in measurements. Testing was performed with all four sets of equipment and differences were not detected. Although this type of equipment is portable, we did not use this function in a door-to-door strategy, due to human resources (only four nutritionists) and logistic reasons, including the availability of electricity in each house (paid by inhabitants), privacy and protection of the information in a suitable space, and also the risk of theft within some areas of the city. Other important reasons, described among others by Dehghan and Merchant [63], are the influence of environment factors (e.g., ambient temperature) in bioelectrical impedance. These authors summarised other factors that can impact in bioelectrical impedance results and they do not recommend their use in epidemiological studies which involve participants from diverse populations. In our case, the measures were taken under controlled conditions and the homogeneity of the population of this small city seems to be ensured. From the statistical point of view, probably a door-to-door strategy would have provided a larger sample size in this second stage, but on the other hand, the accuracy of the measures would be affected. Thus, we opted for the second option, despite knowing that the sample size would be reduced. In fact only 599/1912 (31.3%) went to the measurement points. Keeping in mind future actions to prevent overweight and obesity, an advantage of this option is the identification of characteristics of the inhabitants that should be considered in future interventions designed specifically for these studied urban units or similar ones.

In terms of the second stage, the results presented in Table 3 show a higher participation of the inhabitants of informal (about 6 times more) and transition (2.6 times more) units compared with formal unit. Women were more likely to visit the nutrition team than men. As the time required to complete this stage was around 30–40 minutes, including going from their house to the nutritional status evaluation site, it is logical that unemployed participants, students and other workers (this category includes the housekeepers) visited the nutrition team more, compared to participants who reported to be working. Another important variable with a particular meaning, was BMI based on self-reported measures which was associated with adherence to the second stage. This association seemed to be stronger than the association between adherence and chronic conditions. This result may suggest a certain awareness of body image issues, probably related to concern about health status.

In the first stage (*n* = 1912), a discrepancy between the percentage of female (64.4%) and male (35.6%) was observed. Perhaps, in the sampling process to locate a man more contacts are needed or men of these urban units do not participate as much as women in health studies. Another study on Santiago Island [64] found even more discrepant values 68.7% vs 31.3%. Conditional on participation in the first stage, in a second stage (*n* = 599), the participation was 75.1% for women and 24.9% for men. As the second stage required participant time, and our teams worked mostly during the day, this difference may also be due to a lower employment rate for women. However, the literature indicates that women are more likely to participate in health studies (e.g., [65]). Nevertheless, there are variations across different contexts, specifically, in Africa, Ati el al [37] highlighted the same trend in a obesity study. The migratory condition of the Cape Verdeans (internal migration between the nine inhabited islands and the external diaspora) constitutes another potential explanatory factor.

Given the bias of the second stage, in which BMI based on self-reported measures influenced participation, it is expected that overall adiposity and abdominal adiposity would be overestimated. On the other hand, BMI calculated from self-reported measures leads to underestimation of overweight and obesity because women (and also men) observed in our study, despite reporting their weight correctly, tend to overestimate their height. This is a common issue reported in literature, varying across different groups (e.g., overweight/obese *vs* healthy/underweight individuals, male *vs* female, younger *vs* older individuals)[66, 67]. In spite of these findings, there is a scarcity of data on African context. According to Puonane et al [68], African women saw themselves less obese compared to Caucasian women. Others studies about the perception of height, weight, and overweight and obesity in different ethnic groups, collected from the SUNSET Study, showed that participants from African origin presented the most negative perceptions [66, 69].

In spite of the aforementioned limitations, through a mix of quantitative and qualitative research methods, this study adds important findings regarding an understudied African country and provides new insights for development of future research.

### Conclusions

Our findings indicated that residents of the informal unit and women are the most disadvantaged groups in terms of health outcomes and socioeconomic variables. Focus group discussions and the intensive fieldwork reinforced the higher participation of residents of the informal unit and women in all stages, suggesting the practicability of health promotion campaigns, taking into account the potential of social capital of the informal settlements and the role of the woman in family and society in Cape Verde. The qualitative study also helped us to understand how this particular context can be used to involve women in health promotion in their neighbourhoods, taking into account their decision-making power, their multi-dimensional role in purchasing, processing and preparing food as the pillar of familial food security and also their contributing via non-formal economic activities for their families. On the other hand, the built environment and insecurity of the neighbourhood/urban unit are also modifiable factors in order to promote healthy lifestyles (e.g., physical activity).

The role of urban planners and developers, in planning and/or modifying the built environment, to improve populations’ health and reduce harmful exposures is still not well established in Africa. The complexity and specificity of the dynamic of each city highlight the importance of local studies to bring informal settlements into health statistics and to promote better health in these poorest communities. The link between health planning, urban planning and security of the city needs to be reinforced to minimize health, social and gender inequalities.

## References

[1] Kjellstrom T, Mercado S. Towards action on social determinants for health equity in urban settings. Environment & Urbanization. 2008;20(2):551–574. 10.1177/0956247808096128

[2] Barten F, Mitlin D, Mulholland C, Hardoy A, Stern R. Integrated Approaches to Address the Social Determinants of Health for Reducing Health Inequity. Journal of Urban Health: Bulletin of the New York Academy of Medicine. 2007;84:i164–i173. 10.1007/s11524-007-9173-7 17393340 PMC1892526

[3] Abrahams Z, Mchiza Z, Steyn P. Diet and mortality rates in Sub-Saharan Africa: Stages in the nutrition transition. BMC Public Health. 2011;11:801–819. 10.1186/1471-2458-11-801 21995618 PMC3209469

[4] Vlahov D, Freudenberg N, Proietti F, Ompad D, Quinn A, Nandi V, et al Urban as a determinant of health. Journal of Urban Health. 2007;84:6–26. 10.1007/s11524-007-9169-3 PMC1891649 17356903

[5] Unit Nations. The Millennium Development Goals Reports. New York: Unit Nations; 2012.

[6] WHO, UN Habitat. Hidden cities: Unmasking and overcoming health inequities in urban settings. World Health Organization, The WHO Centre for Health Development, Kobe, and United Nations Human Settlements Programme (UN-HABITAT); 2010.

[7] WHO. Our cities, our health, our future. Acting on social determinants for health equity in urban settings. Report to the WHO Commission on Social Determinants of Health from the Knowledge Network on Urban Settings; 2008.

[8] Unger A, Riley L. Slum health: From understanding to action. PLoS Med. 2007;4(10):e295 10.1371/journal.pmed.0040295 PMC2039756 17958462

[9] Diez-Roux AV. Neighborhoods and health: Where are we and were do we go from here? Rev Epidemiol Sante Publique. 2007;55(1):13–21. 10.1016/j.respe.2006.12.003 17320330 PMC1906739

[10] Corburn J. Healthy City Planning: From Neighbourhood to National Health Equity. Planning, History and Environment Series. Routledge; 2013.

[11] Durand CP, Andalib M, Dunton GF, Wolch J, Pentz MA. A systematic review of built environment factors related to physical activity and obesity risk: implications for smart growth urban planning. Obesity Reviews. 2011;12:e173–e182. 10.1111/j.1467-789X.2010.00826.x 21348918 PMC3079793

[12] Northridge ME, Freeman L. Urban Planning and Health Equity. Journal of Urban Health. 2011;88:582–597. 10.1007/s11524-011-9558-5 21365355 PMC3126931

[13] Sallis JF, Floyd MF, Rodríguez DA, Saelens BE. Role of Built Environments in Physical Activity, Obesity, and Cardiovascular Disease. Circulation. 2012;125:729–737. 10.1161/CIRCULATIONAHA.110.969022 22311885 PMC3315587

[14] Ngau P. For town and country a new approach to urban planning in Kenya. Africa Research Institute; 2013.

[15] Ziraba A, Fotso J, Ochako R. Overweight and obesity in urban Africa: A problem of the rich or the poor? BMC Public Health. 2009;8:465–473. 10.1186/1471-2458-9-465 PMC2803188 20003478

[16] Malhotra R, Hoyo C, Østbye T, Hughes G, Schwartz D, Tsolekile L, et al Determinants of Obesity in an Urban Township of South Africa. South African Journal of Clinical Nutrition. 2008;21:315–320.

[17] WHO. NCD Country Profiles. WHO; 2014.

[18] INE CV. População e Condição de Vida; 2010 INE, Cabo Verde.

[19] Tomita A, Burns J. A multilevel analysis of association between neighborhood social capital and depression: Evidence from the first South African National Income Dynamics Study. J Affect Disord. 2013;144:101–105. 10.1016/j.jad.2012.05.066 22858263 PMC3513630

[20] Carpiano R. Actual or potential neighborhood resources and access to them: testing hypotheses of social capital for the health of female caregivers. Social Science and Medicine. 2008;67:568–582. 10.1016/j.socscimed.2008.04.017 18547699

[21] Corburn J, Cohen A. Why We Need Urban Health Equity Indicators: Integrating Science, Policy, and Community. PLoS Med. 2012;9(8):e1001285 10.1371/journal.pmed.1001285 22904689 PMC3419162

[22] Leyden KM. Social Capital and the Built Environment: The Importance of Walkable Neighborhoods. Am J Public Health. 2003;93:1546–1551. 10.2105/AJPH.93.9.1546 12948978 PMC1448008

[23] Åkesson L, Carling J, Drotbohm H. Mobility, Moralities and Motherhood: Navigating the Contingencies of Cape Verdean Lives. Journal of Ethnic and Migration Studies. 2012;38:237–260. 10.1080/1369183X.2012.646420

[24] Drotbohm H. Horizons of long-distance intimacies: reciprocity, contribution and disjuncture in Cape Verde. History of the Family. 2009;14(2):132–149. 10.1016/j.hisfam.2009.02.002

[25] Marmot M, Friel S, Bell R, Houweling TAJ, Taylor S. Closing the gap in a generation: health equity through action on the social detminants of health. The Lancet. 2008;372:1661–69. 10.1016/S0140-6736(08)61690-6 18994664

[26] Ompad DC, Galea S, Caiaffa WT, Vlahov D. Social Determinants of the Health of Urban Populations: Methodologic Considerations. Journal of Urban Health. 2007;84(1):i42–i53. 10.1007/s11524-007-9168-4 17458704 PMC1891644

[27] Amado M, Gonçalves L, Craveiro I, Cabral J, Lapao L, Simoes R, et al UPHI-STAT—Relatório I, Projecto Planeamento Urbano e Desigualdades em Saúde passando das estatísticas macro para as micro. PTDC/ATP-EUR/5074/2012. IHMT and FCT—Universidade Nova de Lisboa; 2014.

[28] Amado M, Poggi F. Solar energy integration in urban planning: GUUD model. Energy Procidea. Energy Procedia. 2014;50:i277–i284. 10.1016/j.egypro.2014.06.034

[29] Fosgate GT. Practical sample size calculations for surveillance and diagnostic investigations. J Vet Diagn Invest. 2009;21:3–14. 10.1177/104063870902100102 19139495

[30] Gonçalves L, de Oliveira MR, Pascoal C, Pires A. Sample size for estimating a binomial proportion: comparison of different methods. Journal of Applied Satistics. 2012;39(11):2453–2473. 10.1080/02664763.2012.713919

[31] WHO. NCD Country Profiles. WHO; 2011.

[32] Madeira SLR. Towards an Annotated Bibliography of Restructured Portuguese in Africa. Faculdade de Letras da Universidade de Coimbra; 2008.

[33] WHO. Obesity: Preventing and managing the global epidemic. WHO; 2004. 11234459

[34] Gallagher D, Heymsfield S, Heo M, Jebb S, Murgatroyd P, Sakamoto Y. Healthy percentage body fat ranges: an approach for developing guidelines based on body mass index. Am J Clin Nutr. 2000;72:i694–i701. 10.1093/ajcn/72.3.694 10966886

[35] Gòmez-Ambrosi J, Silva C, Galofre J, Escalada J, Santos S, Millán D, et al Body mass index classification misses subjects with increased cardiometabolic risk factors related to elevated adiposity. Int J Obes. 2012;36:i286–i294. 10.1038/ijo.2011.100 21587201

[36] WHO. Waist circumference and waist–hip ratio: report of a WHO expert consultation. WHO; 2008.

[37] Ati JE, Traissac P, Delpeuch F, Aounallah-Skhiri H, Beji C, Eymard-Duvernay S, et al Gender Obesity Inequities Are Huge but Differ Greatly According to Environment and Socio-Economics in a North African Setting: A National Cross-Sectional Study in Tunisia. PLoS ONE. 2012;7(10):e48153 10.1371/journal.pone.0048153 23118943 PMC3485235

[38] Ware L, Rennie K, Kruger H, Kruger I, Greeff M, Fourie C, et al Evaluation of waist-to-height ratio to predict 5 year cardiometabolic risk in sub-Saharan African adults. Nutr Metab Cardiovas. 2014;24:i90–i907. 10.1016/j.numecd.2014.02.005 24675009

[39] Brown LD, Cai TT, DasGupta A. Interval estimation for a binomial proportion. Statistical Science. 2001;16:101–133. 10.1214/ss/1009213285

[40] Lang Z, Reiczigel J. Confidence limits for prevalence of disease adjusted for estimated sensitivity and specificity. Preventive Veterinary Medicine. 2014;113:13–22. 10.1016/j.prevetmed.2013.09.015 24416798

[41] Reiczigel J, Foldi J, Ozsvari L. Exact confidence limits for prevalence of a disease with an imperfect diagnostic test. Epidemiol Infect. 2010;138:1674–1678. 10.1017/S0950268810000385 20196903

[42] R Development Core Team. R: A Language and Environment for Statistical Computing. Vienna, Austria; 2011 Http://www.R-project.org

[43] Sergeant, ESG. Epitools epidemiological calculators. AusVet Animal Health Services and Australian Biosecurity Cooperative Research Centre for Emerging Infectious Disease; 2015.

[44] Escritorio Regional Africano–OMS. Estrategia de cooperação da Organização Mundial da Saúde com os Países 2008–2013, Cabo Verde. Escritorio Regional Africano da OMS; 2009.

[45] Azevedo V. Análise dos factores de risco em Cabo Verde; 2009. Primeiras Jornadas Lusofonas de Cardiologia, Sociedade Portuguesa de Cardiologia.

[46] Doulougou B, Kouanda S, Rossier C, Soura A, Zunzunegui MV. Differences in hypertension between informal and formal areas of Ouagadougou, a Sub-Saharan African City. BMC Public Health. 2014;14(893):i1–i9. 10.1186/1471-2458-14-893 PMC4161842 25175061

[47] Kanter R, Caballero B. Global Gender Disparities in Obesity: A Review. Advances Nutrition. 2012;3:491–498. 10.3945/an.112.002063 PMC3649717 22797984

[48] Abubakari A, Lauder W, Agyemang C, Jones M, KrikB A. Prevalence and time trends in obesity among adult west African population: a meta-analysis. Obes Rev. 2008;9(4):i297–i311. 10.1111/j.1467-789X.2007.00462.x 18179616

[49] Crowther N, Norris S. The current waist circumference cut point used for the diagnosis of metabolic syndrome in sub-Saharan African women is not appropriate. PLoS One. 2012;7(11):ie48883 10.1371/journal.pone.0048883 PMC3493601 23145009

[50] Popkin B, Adair L, SW N. Global nutrition transition and the pandeminc obesity in developing countries. Nutr Rev. 2012;70(1):i3–i21. 10.1111/j.1753-4887.2011.00456.x PMC3257829 22221213

[51] Molata A, Pirie F, Esterhuizen T, Omar M. The prevalence of metabolic syndrome and determination of the optimal waist circumference cutoff points in a rural South African Community. Diabetes Care. 2014;34:i1032–i1037. 10.2337/dc10-1921 PMC3064018 21330644

[52] Moges B, Amare B, Fantahum B, Kassu A. High prevalence of overweight, obesity, and hypertension with increased risk to cardiovascular disorders among adults in Northwest Ethiopia: a cross sectional study. BMC Cardiovascular Disorders. 2014;14(155):i2–i10. 10.1186/1471-2261-14-155 PMC4228065 25373922

[53] Ng M, Fleming T, Robinson M, Thomson B, Graetz N, Margana C, et al Global, regional and national prevalence of overweight and obesity in children and adults during 1980–2013: a systematic analysis for the global burden of disease study 2013. Lancet. 2014;384:i766–i781. 10.1016/S0140-6736(14)60460-8 PMC4624264 24880830

[54] Oyeyemi AL, Adegoke BO, Oyeyemi AY, Deforche B, Bourdeaudhuij ID, Sallis JF. Environmental factors associated with overweight among adults in Nigeria. International Journal of Behavioral Nutrition and Physical Activity. 2012;9(32):1–9. 22452904 10.1186/1479-5868-9-32 PMC3331819

[55] Cook I. Debate. How should steps per day be reported—a proposal using data from Africa. International Journal of Behavioral Nutrition and Physical Activity. 2012;9:7 10.1186/1479-5868-9-7 22309900 PMC3325886

[56] Pillay JD, van der Ploeg HP, Kolbe-Alexander TL, Proper KI, van Stralen M, Tomaz SA, et al The association between daily steps and health, and the mediating role of body composition: a pedometer-based, cross-sectional study in an employed South African population. BMC Public Health. 2015;15:174 10.1186/s12889-015-1381-6 25885183 PMC4344772

[57] Zoettl PA. *Morabeza*, cash or body: Prison, violence and the state in Praia, Cape Verde. International Journal of Cultural Studies. 2014;18:1–16.

[58] United Nations—Office on Drugs and Crime. Study on crime and corruption in Cape Verde. United Nations and Ministry of Justice—Cape Verde; 2007.

[59] McGinn A, Evenson K, Herring A, Huston S, Rodriguez D. The association of perceived and objectively measured crime with physical activity: a cross-sectional analysis. J Phys Act Health. 2008;5(1):117–131. 18209258 10.1123/jpah.5.1.117 PMC4950861

[60] de Almeida Mendes M, da Silva ICM, Hallal PC, Tomasi E. Physical Activity and Perceived Insecurity from Crime in Adults: A Population-Based Study. PloS One. 2014;9(9):e108136 10.1371/journal.pone.0108136 25250805 PMC4176964

[61] UN-Habitat. THE STATE OF AFRICAN CITIES 2014—Re-imagining sustainable urban transitions. United Nations Human Settlements Programme (UN-Habitat); 2014.

[62] Wan SC, Ward CL, Halim J, Gow ML, Ho M, Briody JN, et al Bioelectrical impedance analysis to estimate body composition, and change in adiposity, in overweight and obese adolescents: comparison with dual-energy x-ray absorptiometry. BMC Pediatrics. 2014;14:249–259. 10.1186/1471-2431-14-249 25280868 PMC4288657

[63] Dehghan M, Merchant AT. Is bioelectrical impedance accurate for use in large epidemiological studies? Nutrition Journal. 2008;7:26–31. 10.1186/1475-2891-7-26 18778488 PMC2543039

[64] Rodrigues L, Reis PD. Conhecimentos, Atitudes e Práticas sobre o Paludismo em Cabo Verde. The Global Fund to Fight AIDS, Tuberculosis and Malaria, Ministério de Saúde de Cabo Verde; 2013.

[65] Galea S, Tracy M. Participation Rates in Epidemiologic Studies. Ann Epidemiol. 2007;17:643–653. 10.1016/j.annepidem.2007.03.013 17553702

[66] Nicolaou M, van Valkengoed I, Doak C, van Dam R, Stronks K, Seidell J. Ethnic differences in self-rated overweight and association with reporting weight loss action: the SUNSET study. Eur J Public Health. 2012;22(6):i859–i863. 10.1093/eurpub/ckr155 22051682

[67] Bowring AL, Peeters A, Freak-Poli R, Lim MS, Gouillou M, Hellard M. Measuring the accuracy of self-reported height and weight in a community-based sample of young people. BMC Medical Research Methodology. 2012;12(175):i859–i863. 10.1186/1471-2288-12-175 PMC3561081 23170838

[68] Puoane T, Steyn K, Bradshaw D, Laubscher R, Fourie J, Lambert V, et al Obesity in South Africa: The South African demographic and health survey. Obesity Research. 2002;10:i1038–i1048. 10.1038/oby.2002.141 12376585

[69] Dorsey R, Eberhardr M, Ogden C. Racial/ethnic differences in weight perception. Obesity. 2009;17(4):i790–i795. 10.1038/oby.2008.603 19148119

